# Stellite-6/(WC+TiC) Composite Coatings Produced by Laser Alloying on S355 Steel

**DOI:** 10.3390/ma16145000

**Published:** 2023-07-14

**Authors:** Dariusz Bartkowski, Aneta Bartkowska, Joanna Olszewska, Damian Przestacki, Dariusz Ulbrich

**Affiliations:** 1 Institute of Material Technology, Faculty of Mechanical Engineering, Poznan University of Technology, Piotrowo 3, 61-138 Poznan, Poland; 2 Institute of Material Science and Engineering, Faculty of Materials Engineering and Technical Physics, Poznan University of Technology, Jana Pawła II 24, 61-138 Poznan, Poland; aneta.bartkowska@put.poznan.pl (A.B.); joanna.b.olszewska@student.put.poznan.pl (J.O.); 3 Institute of Mechanical Technology, Faculty of Mechanical Engineering, Poznan University of Technology, Piotrowo 3, 61-138 Poznan, Poland; damian.przestacki@put.poznan.pl; 4 Faculty of Civil and Transport Engineering, Poznan University of Technology, Marii Sklodowskiej-Curie 5 sq., 60-965 Poznan, Poland; dariusz.ulbrich@put.poznan.pl

**Keywords:** WC, TiC, carbides coating, microstructure, microhardness, wear resistance

## Abstract

The paper presents study results of Stellite-6/(WC+TiC) coatings produced by laser alloying with varying contents of reinforcing phases (40%wt and 60%wt content of mixture WC+TiC). The coatings were produced on S355 steel using different laser beam power densities: 76 kW/cm^2^, 115 kW/cm^2^ and 153 kW/cm^2^. The coatings obtained were subjected to microhardness measurements, wear resistance tests, chemical composition analysis and microstructure observations using light microscopy and scanning electron microscopy. It was found that both types of coatings were characterized by higher microhardness and wear resistance in comparison to substrate material. The rate of solidification had an impact on the obtained results of the study.

## 1. Introduction

When it comes to the surface modification of materials in recent years, a great deal of attention has been directed to the use of high-energy heat sources, such as laser radiation in order to change surface layer properties [1,2]. Laser processing is based on a laser beam acting as a source of heat which is used to interfere with the material. Its popularity is due to features such as its limited amount of heat introduced into the material and thus reduced size of the heat-affected zone. A laser beam is also characterized by very fast heating and cooling rates of the material and, as a result of which, it is possible to obtain non-equilibrium structures as well as a very good metallurgical bonding of the produced coating with the substrate [3,4,5]. In this process, binder bound powder materials are frequently used and pre-applied to the substrate which, when remelted, form a coating with improved wear resistance, high hardness and corrosion resistance [3]. Thus, it is possible to improve the mechanical properties of the top layer and extend the life of many materials used for machine parts and tools exposed to severe working conditions in corrosive environments and to high frictional wear. The range of materials that can be used in the alloying process is extensive. Those commonly known include iron alloys [6] as well as high entropy alloys such as AlCoCrFeNi [7], nickel and nickel alloy [8,9] or cobalt alloys. Among the cobalt alloys, the Stellite-6 are of unceasing interest.

This alloy is characterized by high corrosion resistance, friction wear resistance, and good properties at elevated temperatures; however, its wear resistance and hardness in demanding applications may not be satisfactory. One way to improve these properties is to add high hardness and wear resistance particles such as tungsten [10,11,12,13,14,15,16,17,18] and titanium [19,20,21,22,23] carbides to the powder and others [24,25,26]. As a result of remelting the mixture of these materials, a composite layer can be obtained in which carbides are reinforcing particles distributed in the alloy matrix. Here, the volume ratio of carbides and Stellite determines the obtained structure and coating properties. Using various compositions of powder materials, selecting appropriate process parameters such as ex. g. laser beam power density may lead to obtaining coatings of exceptional properties.

Among various surface treatments, surface laser alloying is a commonly used method because it provides high efficiency and good bonding to the substrate. In addition, this method limits the amount of heat introduced into the material. Rapid alloy cooling, which takes place in laser melting and selective laser melting, in addition to allowing the formation of a fine crystal structure, also allows highly saturated solutions, metastable phases and even amorphous structures to be obtained. Therefore, the strengthening of the remelted layer results from the dissolution of foreign atoms and phase transformations taking place, as well as grain fragmentation. Unique properties of the coatings obtained with this method allow adjusting material surfaces for increased wear, corrosion and oxidation resistance. At the same time, laser alloying allows the maintenance of beneficial properties of the material core, such as ductility and strength. In addition, processes such as laser alloying often reduce costs, offering an alternative to time-consuming and energy-intensive diffusion saturation processes, notably also with pertinence to ecology. Moreover, a more expensive material with better properties is located only on the surface of the element, i.e., where it is most exposed to wear and the direct operation of external factors. In tool making, low-carbon steel is often readily available given its low cost, relatively good strength, and ease of forming and joining with other materials. Using laser alloying for this type of material enables the compensation of shortcomings such as low corrosion and wear resistance, which in turn bring about material and financial savings as well as increasing the efficiency of machine or tool parts. 

In the paper of Zhong M. et al. [11], microstructural changes that occurred in welding low-carbon Stellite-6 steel with the addition of varying amounts of tungsten carbide were analyzed. A high-energy CO_2_ laser was used to produce the coatings. In the coatings with a carbide content of 0 to 36%, the presence of a dendritic structure with interdendritic eutectic areas was demonstrated. Here, the tungsten carbide was completely remelted. As for coatings containing carbide from 45 to 100%, the structure was characterized by faceted block-shaped dendrites resembling a flower, butterfly or star. Tungsten carbide particles were mostly remelted and dissolved in the melting area. The dendrite volume increased along with increasing carbide content in the alloying material. Analyses of the obtained properties of coatings made of Stellite-6 powder with the addition of tungsten carbide, produced at various laser powers, were carried out in [15]. Greater remelting depth was characteristic for coatings produced at higher laser powers, whereas the highest hardness was obtained for coatings produced with 60% WC using laser powers of 400 W and 550 W. The average microhardness of these coatings in the remelted zone was approximately 850 HV0.05 and 750 HV0.05. The wear resistance test showed an increased resistance of the coatings produced from the powder with 60% carbide in comparison to the coating produced from pure Stellite-6 powder. 

Laser power used in the alloying process results in laser beam power density on the surface of its operation. This density is so high in the alloying processes that it leads to selective remelting of the alloying material and the substrate in a small area. Throughout the laser alloying process, due to interaction of the beam with the native material and with the coating material, a liquid metal pool is formed. As a result of the heat supplied in the process and convection and gravity movements, as well as the pressure of the laser beam in the pool, the substrate and alloying material are mixed which allows for a permanent blend of both materials. The surface layer formed as a result of laser alloying is often composite in nature and its properties, as well as the chemical and phase composition, remain different from the properties of the substrate material and of the alloying material.

In this paper, the influence of laser beam power density and the composition of alloying materials as the main factors influencing the obtained structure and properties of Stellite-6/(WC+TiC) coatings were tested.

## 2. Materials and Methods

### 2.1. Materials

**Figure 1:**
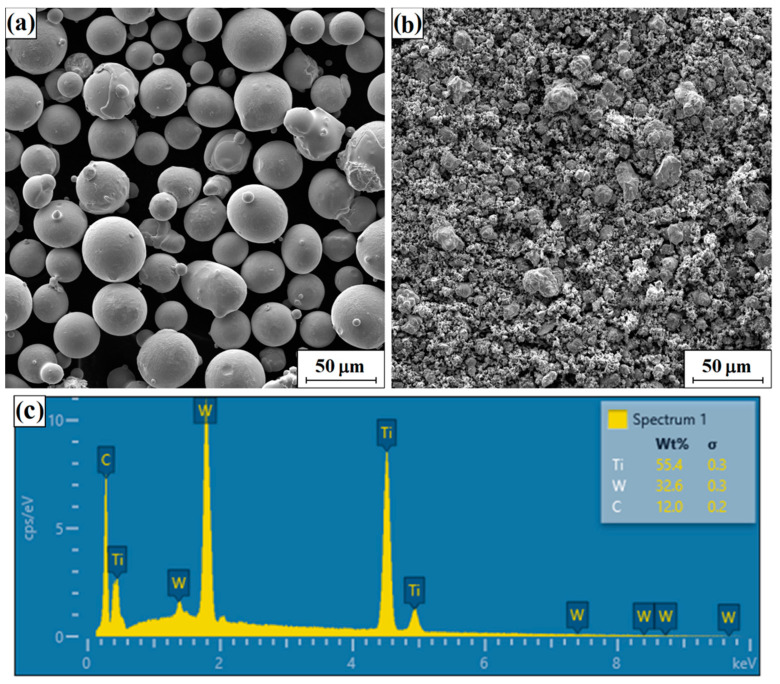
Powder morphology of Stellite-6 (**a**), WC+TiC (**b**) and EDS spectrum for WC+TiC (**c**).

**Figure 2:**
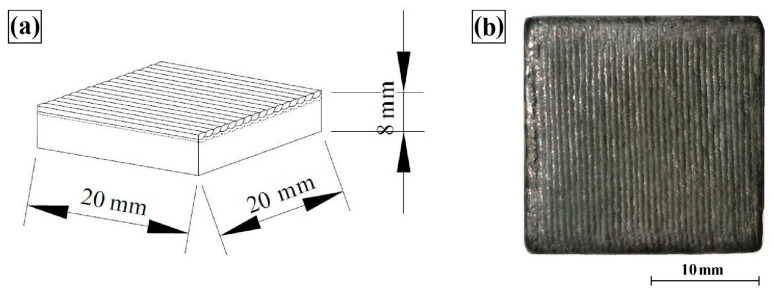
Dimensions of the specimen (**a**) and the view of the specimen after laser processing (**b**).

Surface layers were made on specimens of S355 non-alloy structural steel. Table 1 summarizes the chemical composition of S355 steel and Stellite-6 cobalt alloy. In this study, Stellite-6 powders, tungsten carbides and titanium carbides from Sigma-Aldrich, USA, were used; their morphologies are shown in Figure 1a,b, respectively. Additionally, Figure 1c shows the spectrum for tungsten carbide and titanium powder. The dimensions of each specimen tested are shown in Figure 2a, while an example macroscopic view of the specimens post-laser treatment is shown in Figure 2b.

### 2.2. Laser Alloying

Stellite-6/WC+TiC coatings were produced with varying volume fractions of carbides using different laser beam powers of 600 W, 900 W and 1200 W, as presented in Table 2. Laser beam power density q was calculated from the commonly known formula. The power density (irradiance) is a ratio of power (P) in Watt (W) to the cross-section area of the diameter of the laser beam [q = W/cm^2^]. In research, the laser beam power diameter was 1 mm.

Prior to the alloying process, a 100 µm thick coating consisting of a mixed Stellite-6 powder, WC+TiC powder, water glass and distilled water was applied to the cleaned steel substrate in order to bind the powder materials and maintain adhesion to the substrate. In order for the components of the applied coating to be evenly mixed prior to their application, they were placed in an ultrasonic washer for 15 min. Then, the coating was applied and after the pre-coat dried, the laser beam alloying process was started. The process laser beam scanning speed of v = 3 m/min was used with a constant beam diameter with a circular cross-section of *d* = 1 mm. The distance between the axes of adjacent tracks was *f* = 0.5 mm, while track overlapping *O* resulting from the displacement of the laser source track calculated according to Formula (1) was 50%.

(1) $$O=\frac{d-f}{d}\cdot 100\%$$

**Figure 3:**
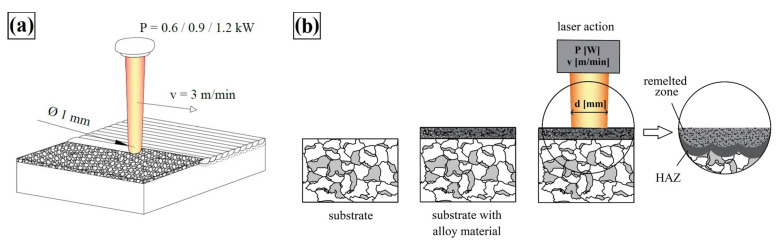
Scheme of laser processing (**a**) and a cross-section through the produced coating (**b**).

**Figure 4:**
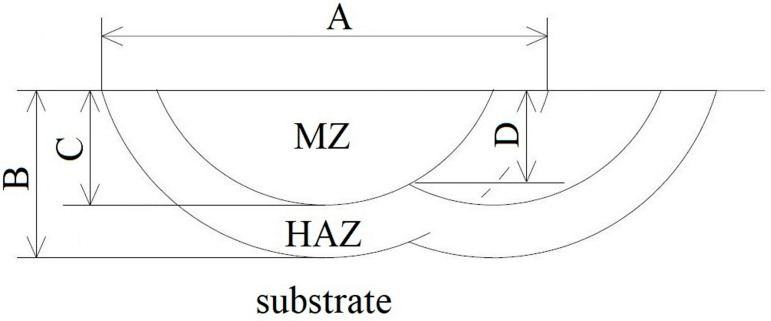
Cross-section of the laser track—basic zones and their measurement; A—width of the laser track, B—depth of the laser track (MZ—remelting zone + HAZ—heat affected zone), C—depth of the remelting zone, D—effective thickness of the produced layer.

Such parameter selection ensures the remelting of previously generated tracks which increases material mixing in the liquid metal pool which is a desirable phenomenon as it leads to homogenization of the remelted zone. In addition, it affects the quenching of previously hardened areas adjacent to the melting zone, reducing the stresses accumulated in these areas. A schematic of laser treatment carried out as well as the cross-section through the coating is shown in Figure 3. The process was carried out using a TRUDIODE 3006 high-power diode laser from TRUDIODE with a rated power of 3 kW which is integrated with the KUKA manipulator that enables automated processing. Figure 4 shows a laser track cross-section on which basic zones and their dimensions are marked.

### 2.3. Characterization of Research Methods

The object under observation was the microstructure of the surface layer produced in the process and the transition of this layer to the core on the cross-section of the analyzed specimen. The test specimens were cut perpendicular to the coating, incorporated in resin and then sanded with sandpaper with increasing gradation from 120 to 2000; then, they were polished with diamond slurry and Al_2_O_3_. Metallographic specimens were digested with a 5% alcohol solution of nitric acid. Microstructure observations were performed with a Mira 3 scanning electron microscope from Tescan. Chemical composition tests were carried out by using the Ultim Max 65 X-ray microanalyst equipped (Oxford Instruments, Abingdon, Great Britain) with an EDS detector, by mapping the distribution of chemical elements in the surface layer produced and by making point analyses of the chemical composition.

The purpose of the microhardness test was to determine the microhardness profile of the surface layer on the cross-section of specimens of the alloyed specimens. The study was conducted according to the Vickers method using the FM-810 microhardness tester (Future-Tech, Kawasaki, Japan) equipped with the FT-Zero automatic measurement software. The set load F was 0.4903 N (50 g) and its operating time was 15 s. 

**Figure 5:**
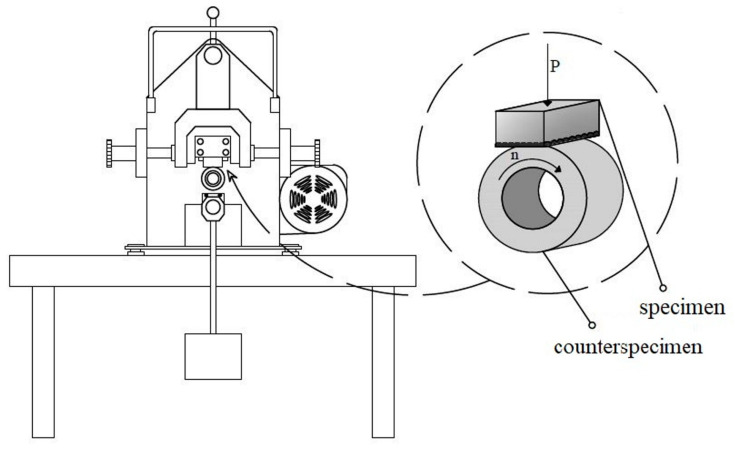
Scheme of the MBT-01 friction machine and the friction pair.

In order to test the friction wear resistance of the produced coatings, the specimens were subjected to tribological wear using the MBT-01 friction machine (Amsler type, Poznan, Poland) at a constant load of F = 98 N. The test was carried out under technical friction conditions—without the addition of lubricant; in such conditions, it is possible to have atmospheric compounds in the frictional couple system, e.g., oxygen causing oxidation of the mating surfaces. It is also possible to find particles detached from the surfaces of the mating elements, affecting abrasive wear advantage in the wear mechanism. The schematic of the device and of the frictional couple is shown in Figure 5. The counter-specimen was C45 steel after the quenching process from 950 °C in water and high tempering at 520 °C, with a hardness of 34 HRC, in the form of a ring set in rotation at a speed of *n* = 250 min^−1^. Wear resistance was determined on the basis of the relative weight loss of the specimen Δm/m_0_ [%] in the time unit t [h]. Weight loss was recorded every half hour using the RADWAG AS60/220/C/2 analytical balance with an accuracy of 0.00001 g. In order to compare the results, S355 steel was also subjected to wear without the alloying process. Observations of the examined surfaces were made using the TESCAN Mira 3 scanning microscope. Element distribution maps using the EDS were also made on the tested surfaces. 

## 3. Results

### 3.1. Microstructure and Chemical Composiion

**Figure 6:**
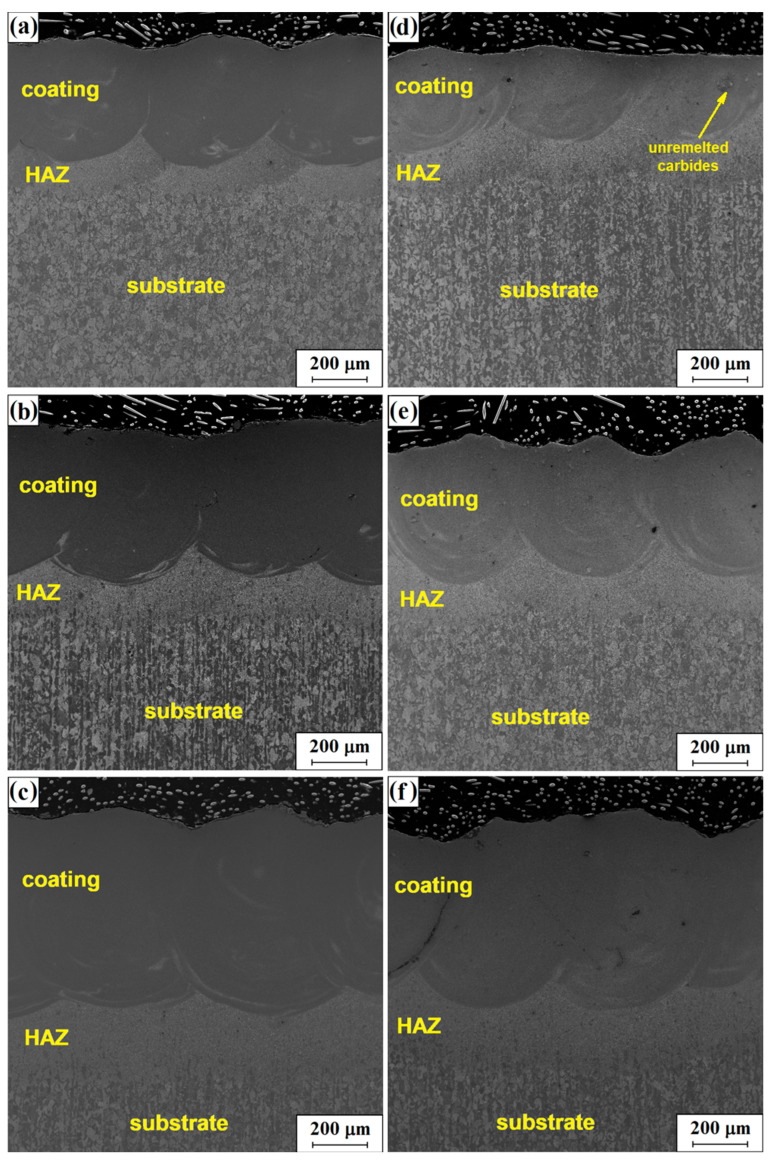
The microstructure of the coatings produced in the laser processing process: Stellite/40% (WC+TiC): 76 kW/cm^2^ (**a**); 115 kW/cm^2^ (**b**); 153 kW/cm^2^ (**c**) and Stellite-6/60% (WC+TiC) for the laser beam power density 76 kW/cm^2^ (**d**), 115 kW/cm^2^ (**e**) and 153 kW/cm^2^ (**f**).

Figure 6 summarizes microstructures of all the coatings. The photos clearly show a structure comprising three characteristic zones as in Figure 4. The first of these is the remelted zone (MZ) near the surface of the specimen, where there were structural changes related to the change in chemical composition in alloying and to the impact of temperature. Another clearly visible zone is the heat-affected zone (HAZ): its chemical composition corresponds to the composition of the substrate material; however, the structure of this area differs from the core. As a result of the increased temperature and rapid cooling at a speed higher than critical, in this zone one track was quenched and then another was tempered. Just below the heat-affected zone, the structure of the core of the surface-treated material can be observed: there are no visible changes in this area and its structure corresponds to the material in the initial state. 

Figure 6 clearly shows an increased depth of the remelting zone along with an increased laser beam power density; this relationship is confirmed by the measurement results listed in Table 3. The increase in remelting depth is associated with a greater amount of heat supplied to the material which contributes to greater substrate remelting.

In Figure 6, individual carbides (bright particles) in the remelted zone that were not remelted can be observed. The example is marked with a yellow arrow. As a result of digestion, it is also possible to observe structure segregation in this zone which is associated with thermal movements in the pool throughout alloying. In certain places of the remelted areas, porosities are visible; however, the degree of porosity in the produced layers is slight. The presence of a small proportion of porosity is due to the presence of bubbles of gases such as oxygen or hydrogen which, due to the high rate of solidification, failed to escape to the surface and were retained in the volume of the material.

Significant dimensions of the produced laser tracks along with the effective depth of the produced layer are summarized in Table 3, giving their average value. The measurements were made on 10 randomly selected laser tracks.

A relationship between laser beam power density used in the process and the resulting depth of the remelting zone can be clearly seen (Table 3, Figure 6). The average depth of the laser tracks obtained increased as the laser power increased. The increase in laser beam power density caused the coating material to melt to greater depths for material with both lower and higher content of WC+TiC carbides. Table 3 summarizes the average effective remelting depth. The effective melting depth is the smallest depth at which the remelted area borders the heat-affected zone. This depth is significant in the case of wear of the produced layer as it is the minimum depth at which the tool is protected against excessive wear. 

**Figure 7:**
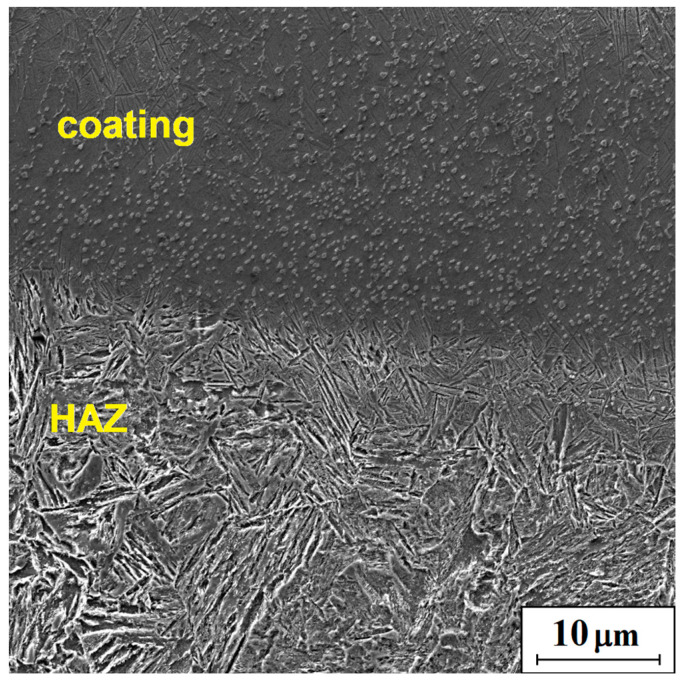
Microstructure of the Stellite-6/40% (WC+TiC) coating (q = 153 kW/cm^2^).

In the transition area between MZ and HAZ, a higher concentration of carbides could be found. A typical picture of the microstructure is shown in Figure 7. Carbide segregation in the vicinity of the heat-affected zone may be associated with convective and gravitational motion occurring in the remelted material throughout the laser beam operation. Movements occurring in a liquid metal pool under the influence of a laser beam are directly related to the fluctuations in the chemical composition. The structure in the area between the carbides, visible close to the HAZ, could have been influenced by the difference between the temperature gradient and the cooling rate in the area close to the HAZ and closer to the surface of the material, which resulted in crystallization of fine dendrites with a cellular structure closer to the HAZ due to faster heat dissipation, unlike column dendrites in higher areas; a similar relationship was observed in laser welding of H13 steel with Stellite-6 [18].

**Figure 8:**
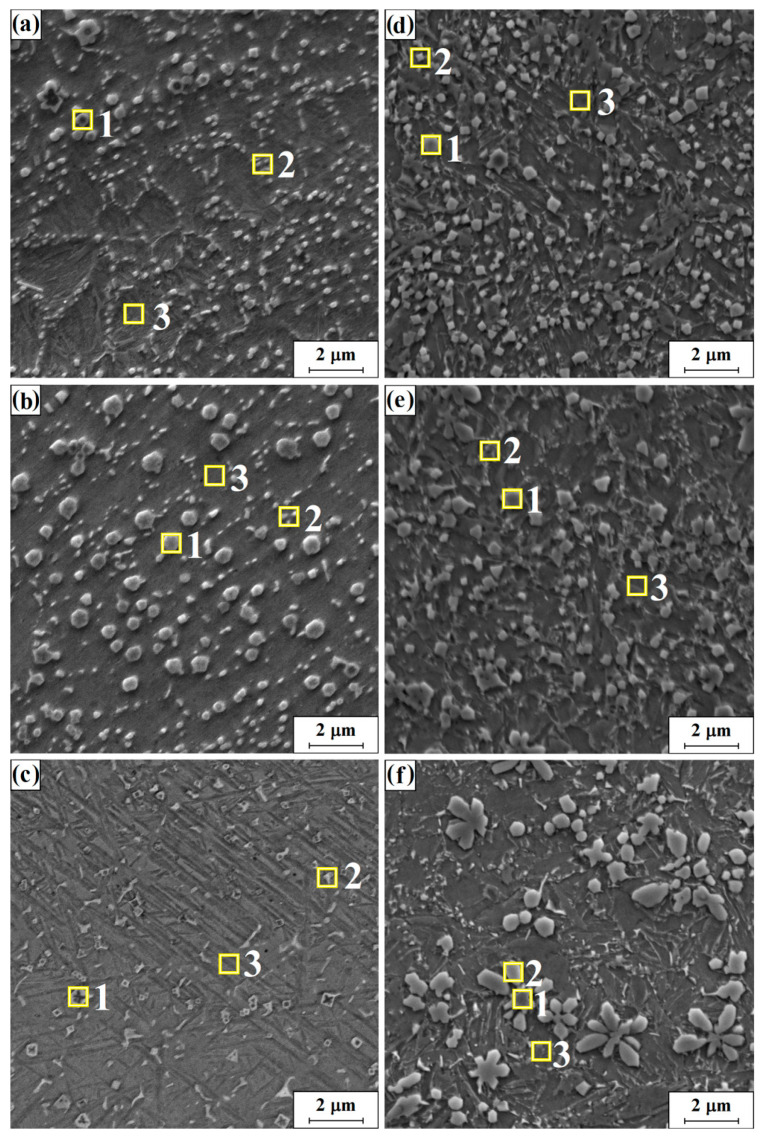
The microstructure of the central area of the remelted zone for the coating: Stellite/40% (WC+TiC): 76 kW/cm^2^ (**a**); 115 kW/cm^2^ (**b**); 153 kW/cm^2^ (**c**) and Stellite-6/60% (WC+TiC) for the laser beam power density 76 kW/cm^2^ (**d**), 115 kW/cm^2^ (**e**) and 153 kW/cm^2^ (**f**).

Figure 8 shows the microstructures of the central area of the remelted zone for the Stellite-6/40% (WC+TiC) and Stellite-6/60% (WC+TiC) coatings produced using different laser beam power densities. For these coatings, the chemical composition analysis was carried out using the EDS method and the measurement sites were marked with yellow squares in the figure. The measurement sites marked the reinforcing phase occurring in the produced surface layer most likely in the form of WC+TiC carbides and matrix. The conducted studies confirmed the occurrence of the reinforcing phase in the form of white precipitations with an increased content of W and Ti. In the matrix, an increased iron content was found, which came from the substrate. The detailed values obtained for the analyzed elements are summarized in Table 4.

Figure 8a shows a Stellite-6/40% (WC+TiC) coating produced by a 76 kW/cm^2^ laser beam. At measuring point 1 located in the area of bright phases, a high concentration of 10% tungsten and 9.6%, titanium as well as the presence of 6.1% carbon are detectable. Figure 8b shows a 40% carbide coating produced by a laser beam with a higher power density of 115 kW/cm^2^. In the EDS analysis of the presented coating, point 1 (Table 4) demonstrated a high concentration of 21.4% tungsten and of 23.8% titanium. In the presented structure, an increased content of cobalt (17.3%) and chromium (6.8%) can be observed at point 3 located in the matrix area. Cobalt and chromium are the main components of the Stellite-6 alloy. In the presented coating, its content in the alloying material is higher which increased the proportion of these elements. Figure 8c shows the Stellite-6/40% (WC+TiC) coating produced by a laser beam with the highest power density. Analysis of point 3 showed a high iron content reaching 72.1% of 13.5% cobalt and 5.6% chromium. In analysis 1, apart from the presence of 39.4% iron, a high concentration of 23.8% titanium,17% tungsten and 9.5% carbon—was also shown.

Based on the obtained EDS results (Table 4), it is possible to determine the approximate phase composition of the studied areas. In the brighter area of phase occurrences 1 and2 (Figure 8d) for the Stellite-6/60% (WC+TiC) coating produced at a power density of 76 kW/cm^2^, a high concentration of approximately 30% titanium was shown as well as approximately 25% of tungsten, approximately 30% of iron, and 15% carbon. This may indicate the presence of complex tungsten and titanium carbides in these areas. The matrix for carbides in site 3 in this coating is a structure with a high proportion of approximately 79% iron, as well as approximately 4% tungsten, approximately 7% carbon and approximately 2% titanium. The resulting structure allows us to state that larger carbides added to the Stellite-6 powder were selectively remelted while some smaller carbides, perhaps due to the high solubility of tungsten carbide in remelted metals, were remelted and mixed with Stellite-6 and the substrate material. At site analysis 3, the presence of approximately 4% cobalt was also demonstrated as well as approximately 2% of chromium and 1.2% nickel. Cobalt and chromium, with a high carbon content, may form precipitates of secondary carbides. Studies have shown that there is a high concentration of light elements including titanium. On the basis of this observation and on literature sources, it can be determined that it is a core-shell phase in which the core is TiC—carbide depleted tungsten, while it is surrounded by (Ti and W) C carbide [27,28]. The hardness of this type of phase is greater than the hardness of WC carbide, which results in increased hardness of structures produced with TiC carbide, allowing for their formation [28]. Figure 8e also shows the microstructure of the coating made of 40% Stellite-6 and 60% powder (WC+TiC) but which was this time produced by a laser beam with a higher power density of 115 kW/cm^2^. Three points were marked on the photo where chemical composition analyses were performed. The sites were selected in the areas of occurrence of separate phases. The results of the EDS analysis (Figure 8e) are presented in the same way as previously. In point 1, the results indicate a high content of approximately 24% titanium and 31% tungsten. Carbon is also present in these areas at approximately 12% as well as iron which is present at approximately 39%. On the basis of the chemical composition, the tested phase can be roughly defined as double carbide (Ti0.88W0.12) C. Theoretical carbon content in the given carbide is 15.72%, of tungsten 28.93% and of titanium 55.34%, which, with the exception of titanium, approximately coincides with the results obtained in the analysis. Points 2 and 3 are located within a phase different from carbides. Composition analysis showed a high concentration of iron in selected places of 69.9% (point 2) and 78.7% (point 3) and the presence of 8.6% and 7.5% carbon, 5.2% and 4.7% cobalt, and 7.2% and 5.2% tungsten. The high iron content indicates a high remelting of the substrate and dilution of the alloying material. In laser alloying, the share of the substrate material in the structure may reach up to 90%. Due to low formation heat, tungsten carbide is easily dissolved in remelted metals, which explains its share in the obtained analysis. The obtained results also indicate a high uniformity of the structure, which can be associated with a high carbide content, especially tungsten carbide, whose thermal conductivity is the highest of all transition metal carbides and impacted very good mixing of the substrate and the alloying material. Figure 8f shows a Stellite-6/60% (WC+TiC) coating produced by a laser beam with the highest power density of 153 kW/cm^2^. Measurement points 1 and 2 are located in the areas of the phase, whose precipitates take characteristic shapes resembling a butterfly, flower or star, which were observed earlier in the literature. The solidification process, which results in the formation of such shapes, explains that during heating some larger carbides can be partially melted and other smaller ones can be completely remelted and then mixed with Stellite-6. When the cooling and solidification process begins, the partially remelted carbide particles initiate heterogeneous crystallization for dendrites of different shapes depending on the local composition and temperature gradient [4]. Measurement points 1 and 2 showed a high content of 41.4% and 36.2% titanium, 31.7% and 26.8% tungsten, 14.7% and 13.8% carbon, and 10.7% and 19.6% iron, respectively. This indicates, as previously, that complex tungsten and titanium carbides may be present in these areas. In point 3 (Figure 8f), a very high iron content of 77.9%, tungsten content of 4.6%, carbon of 6.8% and cobalt of 5.7% were shown. Additionally, in point 3, the presence of 1.8% titanium, 2.3% chromium and a small proportion of nickel (0.8%) were demonstrated. The presence of titanium and tungsten in the obtained result, as before, indicates the remelting and mixing of some of the carbides contained in the starting powder with the Stellite-6 material and the substrate. Comparing the morphology of the obtained structure with the structures obtained at lower laser beam power densities, a significant increase in carbides in relation to lower power can be observed. By comparing the morphology of the obtained structures with the initial powder of WC+TiC carbides, it can be concluded that the carbides were mostly remelted to form the precipitates of secondary carbides formed in the process.

### 3.2. Microharndess

**Figure 9:**
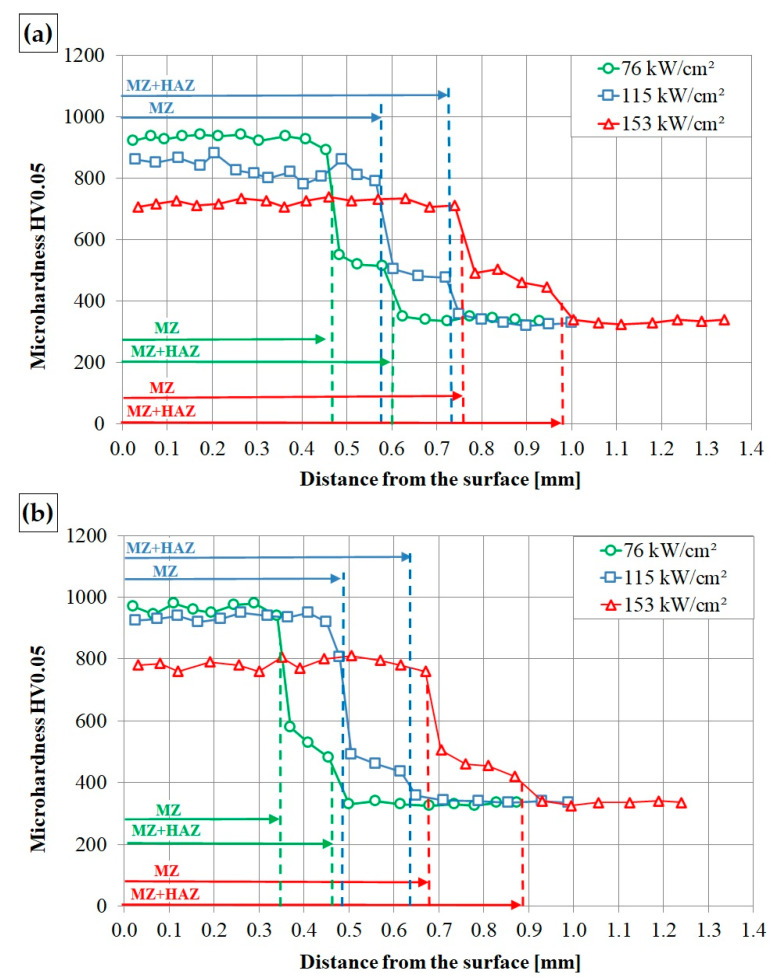
Microhardness profiles for coating used a different laser beam power density: Stellite-6/40% (WC+TiC) (**a**) and Stellite-6/60% (WC+TiC) (**b**).

Microhardness profiles of composite coatings made of powders with varying volume fractions of carbides with increasing laser beam power densities are summarized in Figure 9 which shows the depths of the remelted zones and laser tracks (MZ+HAZ) in the axis at the measurement site. Higher beam power densities affected greater depths of remelting. An increase in the remelting zone is associated with an increase in the degree of dilution ratio (DR), which can be determined from the [29], where:

(2) $$\mathrm{DR}=1\;-\frac{t_{C}}{d_{\mathrm{MZ}}}$$

t_c_ is the thickness of the coating applied prior to the start of laser operation [μm] and d_MZ_ is the average thickness of the remelted zone [μm].

Due to an increase in the degree of dilution ratio, the impact of the substrate on mechanical parameters of the produced coatings is greater, which is associated with an increase in the share of substrate components in the resulting layer. In coatings with a 40% volume fraction of WC+TiC in the initial powder, the highest average value of microhardness amounting to approximately 950 HV0.05 was obtained for the coating produced using the lowest beam power density—76 kW/cm^2^ (Figure 9a). This coating is characterized by an even microhardness along the entire depth of the laser track. Then, its microhardness decreases in the heat affected zone, reaching the value of approximately 480 HV0.05, and finally it decreases to the hardness of the ferritic–pearlitic substrate.

For the coating made of Stellite-6/40% (WC+TiC) powder, performing laser beam alloying with the higher power densities of 115 kW/cm^2^ and 153 kW/cm^2^ resulted in microhardness values of approximately 830 HV0.05 and 700 HV0.05, respectively. The microhardness of the coating has equal values over the entire analyzed cross-section of the laser track. With the increase in the power density of the laser beam, the microhardness decreases which is related to the dissolution of carbides in the matrix composed of both Stellite-6 and the steel substrate material. The hardness of the heat-affected zone with the increase in the laser beam power density slightly decreases to the value of approximately 450 HV0.05. It can also be seen that the microhardness gradient between the remelted zone and the heat flow zone decreases with increasing laser beam power density.

For coatings with a fraction volume of carbides of 60% in the starting material, the highest average microhardness of approximately 1000 HV0.05 was obtained for a coating alloyed with a laser beam power density of 76 kW/cm^2^. Due to the smallest remelting depth, the share of high-hardness coating materials in the produced layer is the largest in this case, which is associated with the presence of hard carbides. The coating concerning 60% of carbides produced by the laser beam with a power density of 115 kW/cm^2^ had an average hardness of approximately 950 HV0.05. For a power density of 153 kW/cm^2^, the average microhardness is approximately 800 HV0.05. (Figure 9b). It is worth mentioning that despite the doubling of the laser beam power density, the obtained coatings have high and uniform microhardness values.

### 3.3. Wear Resistance

Studies of frictional wear resistance were carried out for all analyzed specimens. Admittedly, it was presumably assumed that the most advantageous would be coatings produced at a lower laser beam power. This choice was dictated by the economic aspect of the coating production, considering the relatively high price of mixed WC+TiC carbide as well as the less energy-intensive laser alloying process. The obtained results were compared with the course of wear of S355 steel which is the substrate for the produced coatings.

**Figure 10:**
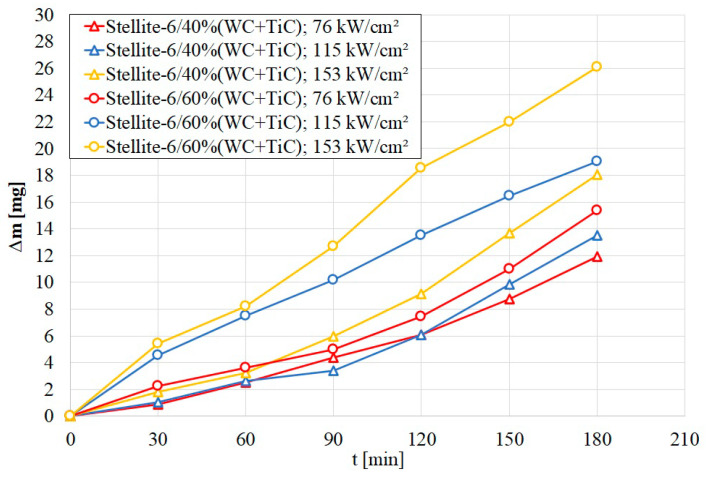
Wear resistance Stellite-6(WC+TiC) coatings.

In the graph showing relative weight loss during wear correlated to test time (Figure 10), for S355 steel a very large influence of the produced coating on the reduction in the specimen mass loss during friction was visible. The specimen from S355 steel had an initial stage of intensive material wear which passed into the fixed wear stage, after which its wear increased again. The mass loss after 180 min for S355 steel was approximately 160 mg. Due to significant differences in the mass loss of the substrate and coatings, the graph of substrate consumption is not attached.

On the other hand, for specimens with a produced coating, a small and uniform loss of mass can be observed during the test without significant changes in its magnitude which would indicate less intensive wear. Analyzing all wear curves, it can be seen that a produced coating containing more carbides does not show significantly increased frictional wear resistance. It can be assumed that too many carbides in relation to the matrix may cause their tearing, which may indicate a lack of good adhesion between the matrix and the reinforcing phase. A significant difference was noted when the highest laser beam power density was used. Then, the worst parameters in terms of frictional wear resistance were observed for the specimen marked Stellite-6/60% (WC+TiC) q = 153 kW/cm^2^ on the graph. The increase in wear was 8 mg in comparison with the coating with a lower carbide content.

**Figure 11:**
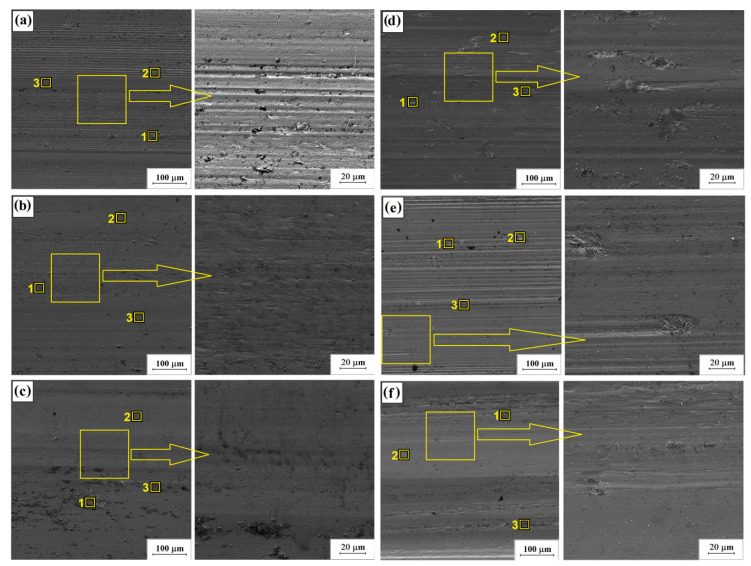
Surface after wear resistance for Stellite/40% (WC+TiC): 76 kW/cm^2^ (**a**); 115 kW/cm^2^ (**b**); 153 kW/cm^2^ (**c**) and Stellite-6/60% (WC+TiC) for the laser beam power density 76 kW/cm^2^ (**d**), 115 kW/cm^2^ (**e**) and 153 kW/cm^2^ (**f**).

Surface areas of the specimens after frictional wear tests are summed up in Figure 11. At a smaller magnification for each drawing, a yellow square was drawn for which the enlarged area is shown in the figure next to it. From this area, chemical composition maps were made using the EDS method. The chemical composition analysis was performed for the marked places numbered 1–3 and the results are presented in Table 5.

For the Stellite-6/40% (WC+TiC) specimen, with a laser beam power density of 76 kW/cm^2^ (11a), typical wear traces in the form of grooves are visible. Whereas, for Stellite-6/60% (WC+TiC) coatings there are clear traces of chipping, which had an impact on the reduction in frictional wear resistance (Figure 11d). Increasing the laser beam power density caused a decrease in wear resistance of both kind of coatings on their surface, of which traces related to surface oxidation in the form of dark precipitates could be observed (Figure 11c,f).

**Figure 12:**
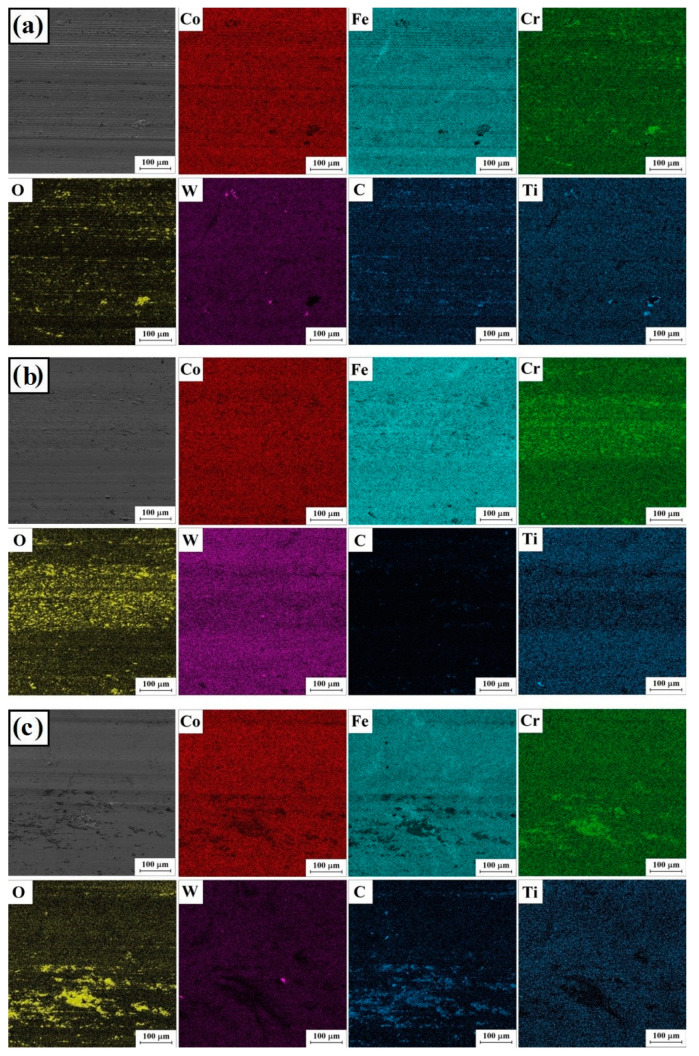
Surface condition after wear resistance tests: Stellite-6/40% (WC+TiC)—76 kW/cm^2^ (**a**), Stellite-6/40% (WC+TiC)—115 kW/cm^2^ (**b**) and Stellite-6/40% (WC+TiC)—153 kW/cm^2^ (**c**).

**Figure 13:**
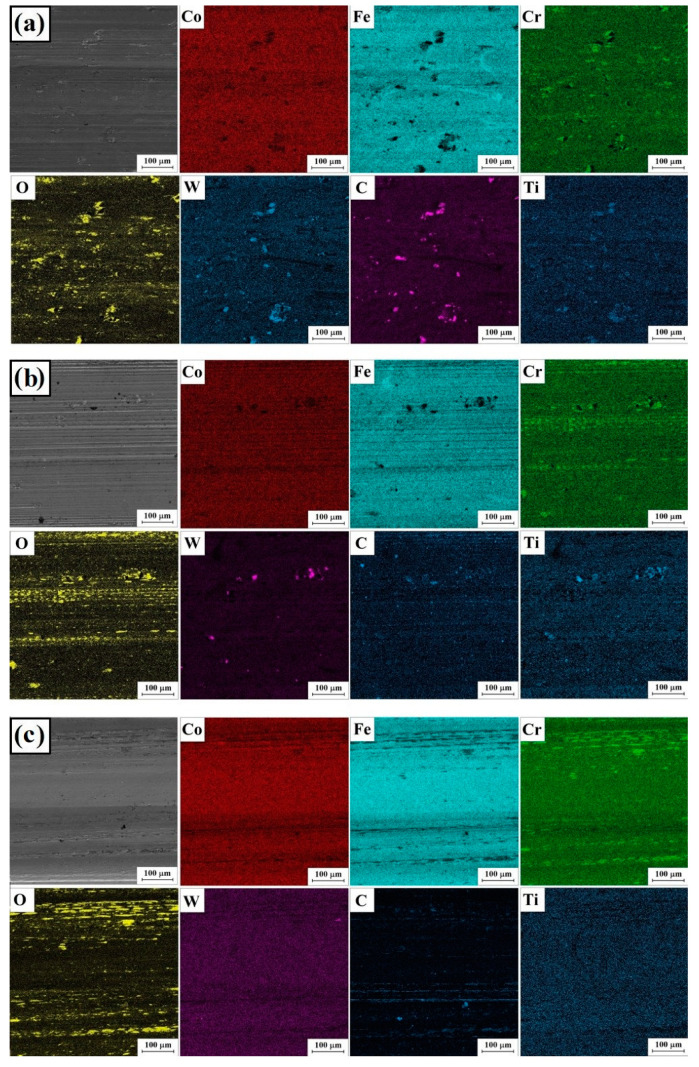
Surface condition after wear resistance tests: Stellite-6/60% (WC+TiC)—76 kW/cm^2^ (**a**), Stellite-6/60% (WC+TiC)—115 kW/cm^2^ (**b**) and Stellite-6/60% (WC+TiC)—153 kW/cm^2^ (**c**).

Figure 12 and Figure 13 show the results of mapping the chemical composition using the EDS method with basic elements included in the composition of the alloyed material (Stellite-6/WC+TiC) and the substrate, which was an iron alloy, as well as oxygen associated with oxidation of the surface caused by abrasion.

The production of coatings, regardless of the carbide share in the alloying material, resulted in an almost 10-fold increase in wear resistance of S355 steel. Considering total weight loss after the test, it can be concluded that the coating with a lower carbide content is characterized by greater wear resistance. The reduction in wear resistance of a coating with 60% share of WC+TiC carbides may have been caused by the fact that the increased carbide content reduced the Stellite-6 share, which, along with the remelted substrate material, constitutes the matrix binding carbides by creating metallurgical and adhesive bonding. In this case, a higher carbide content may have reduced their resistance to chipping from the matrix and the smearing of the counter-specimen on the surface of the C45 steel material, which is a material with higher plasticity. This process could ultimately result in greater wear of the coating material as a result of the presence of hard abrasive grains in the counter-specimen material as well as in various areas. A lower remelting depth of the coating with a 60% share of WC+TiC may also have influenced its lower resistance.

In the SEM photos (Figure 11), there are visible signs of wear due to micro-cutting and grooving under technical dry friction conditions. The occurrence of this abrasive wear mechanism is associated with pulling carbide particles from the matrix during friction, which subsequently act as an abrasive, and their very high hardness compared to the hardness of the matrix promotes its wear. A high oxygen content in the analysis areas indicates a wear mechanism through oxidation. This wear occurs due to the absorption of oxygen present in the atmosphere, which affects the formation of oxides on the friction surfaces, mainly with elements with a high affinity for oxygen, such as iron and chromium. 

## 4. Conclusions

In the tests of Stellite-6/(WC+TiC) coatings produced on S355 steel in laser processing, it was shown that laser alloying with properly selected process parameters enables improvement of the operational properties of workpieces by increasing their wear resistance as well as increasing the surface layer hardness. The structure created as a result of laser alloying was characterized by the presence of three different areas: the remelting zone, the heat-affected zone and the substrate material zone. Depending on the laser processing parameters used, i.e., the laser beam power density, the geometry and structure of the surface layers was changed. The structure, geometry and properties of produced coatings were also influenced by the share of mixtures of tungsten and titanium carbide in the alloying material. On the basis of the conducted research, the following conclusions may be formulated:

1. With an increase in laser beam power density, the depth and width of the remelted zone increased due to a greater amount of heat supplied to the substrate;
2. For both types of coatings, as the laser beam power density increased, the effective thickness of the remelting zone approached the thickness of the remelted zone obtained in the laser track axis;
3. The produced structures were characterized by low porosity and occasionally occurring porosities were caused by the presence of gas bubbles which were retained in the volume of the material by the solidification front;
4. In both types of coatings (regardless of the amount of carbides) the maximum microhardness obtained is similar and is 980 ± 20 HV0.05;
5. During wear resistance tests, the coatings produced at the lowest laser beam power density were analyzed, for specimens with coatings a small and gradual loss of material was observed in the test. The produced coatings were characterized by almost a 10-fold greater resistance to frictional wear in comparison with S355 steel. The coating with a lower carbide proportion showed a slightly higher wear resistance, which may be due to a lower tendency of carbides to chip away from the matrix;
6. Observation of the coating surface subjected to wear test and EDS analysis showed a share of abrasive wear resulting from micro cutting and wear as a result of oxidation, as well as mass losses resulting from carbide chipping.

## Figures and Tables

**Table 1:** Chemical composition of steel substrate and Stellite-6 powder [wt.%].

Type of Material	C	Si	Mn	P	S	Cu	Fe	Cr	W	Ni	Co
S355	0.21	0.51	1.53	0.032	0.031	0.35	rest	-	-	-	-
Stellite-6	1.2	1.81	-	-	-	-	1.75	28.5	4.6	1.52	rest

**Table 2:** Laser processing parameters.

Laser Beam Power P [W]	Laser Beam Power Density q [kW/cm^2^]	Percentage of Powders in the Pre-Coat
600	76	Stellite-6/40% (WC+TiC)
900	115	
1200	153	
600	76	Stellite-6/60% (WC+TiC)
900	115	
1200	153	

**Table 3:** Dimensions of the laser tracks.

Dimensions of Individual Zones of the Laser Track [μm]	Type of Coating					
	Stellite-6/40% (WC+TiC)			Stellite-6/60% (WC+TiC)		
	q [kW/cm^2^]			q [kW/cm^2^]		
	76	115	153	76	115	153
**A** Average width of the laser track	877	986	1009	774	863	1014
**B** Average depth of laser track (MZ+HAZ)	605	747	1007	476	628	886
**C** Average depth of remelting zone	485	584	777	377	483	702
Average depth of HAZ **B**–**C**	120	163	230	99	145	184
**D** Average effective depth of remelting	343	433	694	224	316	555

**Table 4:** Chemical composition by the EDS method for the areas marked in Figure 8 [wt.%].

Designation	No	Fe	C	W	Ti	Co	Cr	Ni
Stellite-6/ 40% (WC+TiC) 76 kW/cm^2^	1	57.6	6.1	10.0	9.6	11.4	4.9	0.5
	2	55.9	6.6	9.4	9.5	11.7	5.4	1.5
	3	72.4	4.9	4.5	2.7	10.7	4.8	0.0
Stellite-6/ 40% (WC+TiC) 115 kW/cm^2^	1	30.5	9.1	21.4	23.8	9.4	4.5	1.3
	2	39.8	8.5	15.5	17.2	12.2	5.5	1.2
	3	66.3	3.6	4.0	1.4	17.3	6.8	0.7
Stellite-6/ 40% (WC+TiC) 153 kW/cm^2^	1	39.4	9.5	17.0	23.8	6.8	3.5	0.0
	2	62.1	6.5	7.8	7.1	11.0	4.2	1.3
	3	72.1	3.8	3.4	1.5	13.5	5.6	0.0
Stellite-6/ 60% (WC+TiC) 76 kW/cm^2^	1	28.1	14.6	25.1	30.3	1.0	1.0	0.1
	2	31.4	14.9	22.7	28.2	1.9	0.9	0.0
	3	78.8	7.1	4.3	2.4	4.4	1.8	1.2
Stellite-6/ 60% (WC+TiC) 115 kW/cm^2^	1	39.0	12.5	30.7	24.1	2.2	1.1	0.4
	2	69.9	8.6	7.2	5.8	5.2	2.7	0.6
	3	78.7	7.5	5.2	1.9	4.7	2.0	0.0
Stellite-6/ 60% (WC+TiC) 153 kW/cm^2^	1	10.7	14.7	31.7	41.4	0.4	0.8	0.3
	2	19.6	13.8	26.8	36.2	1.4	0.9	1.3
	3	77.9	6.8	4.6	1.8	5.7	2.3	0.8

**Table 5:** Chemical composition by the EDS method for the areas marked in Figure 11 [wt.%].

Designation	No	Fe	C	W	Ti	Co	Cr	Ni	O
Stellite-6/ 40% (WC+TiC) 76 kW/cm^2^	1	55.3	3.1	2.2	2.5	5.6	2.3	0.0	29.1
	2	60.9	3.4	5.9	5.0	12.5	5.5	0.0	6.8
	3	52.4	5.2	10.0	8.8	13.7	6.9	0.0	3.1
Stellite-6/ 40% (WC+TiC) 115 kW/cm^2^	1	55.7	9.2	2.3	1.3	6.7	2.8	0.1	21.9
	2	59.8	4.5	7.8	6.0	12.7	4.9	0.0	4.3
	3	49.8	10.4	5.7	4.7	9.1	3.5	0.6	16.2
Stellite-6/ 40% (WC+TiC) 153 kW/cm^2^	1	49.6	13.9	2.4	1.4	4.6	2.4	0.8	24.9
	2	71.2	4.0	4.8	3.8	9.2	4.5	0.0	2.4
	3	72.1	7.6	3.2	3.2	5.4	2.0	0.0	5.6
Stellite-6/ 60% (WC+TiC) 76 kW/cm^2^	1	0.6	6.7	78.1	13.0	0.9	0.0	0.3	0.5
	2	67.5	5.1	9.8	9.8	3.2	1.8	0.0	2.8
	3	55.7	7.9	4.40	3.4	2.6	0.9	0.0	25.1
Stellite-6/ 60% (WC+TiC) 115 kW/cm^2^	1	4.4	6.9	71.7	13.0	0.3	0.1	0.0	3.6
	2	68.4	3.2	3.5	4.6	1.1	0.9	0.0	18.2
	3	14.4	13.4	27.4	29.2	0.0	0.3	0.2	15.2
Stellite-6/ 60% (WC+TiC) 153 kW/cm^2^	1	69.7	5.2	1.0	1.0	1.7	0.4	0.5	20.6
	2	91.7	3.2	1.8	1.4	1.0	0.3	0.0	0.6
	3	56.2	13.9	1.7	1.3	0.2	0.2	1.3	25.2

## Data Availability

Data available on request.
